# Fabry App: the value of a portable technology in recording day-to-day patient monitored information in patients with Fabry disease

**DOI:** 10.1186/s13023-023-02999-6

**Published:** 2024-01-11

**Authors:** Simona D’Amore, Mark Mckie, Andrew Fahey, David Bleloch, Giuseppina Grillo, Michael Hughes, Uma Ramaswami

**Affiliations:** 1 Lysosomal Storage Disorders Unit, Royal Free Hospital NHS Foundation Trust, Pond Street, London, NW3 2QG UK; 2 HealthTouch Ltd, Little Halt, Bull Lane, Chislehurst, Kent, BR7 6NX UK; 3 Present Address: Chiesi Farmaceutici S.P.A., Via Paradigna 131/A, 43122 Parma, Italy

**Keywords:** Fabry disease, Smartphone applications, Patient reported data, Disease monitoring, Disease management

## Abstract

**Background:**

Fabry disease is a rare inherited disorder resulting from deficient α-galactosidase A enzyme activity. Common disease manifestations are sweating abnormalities, neuropathic pain, gastrointestinal symptoms and fatigue. Challenges are faced by health care professionals in evaluating symptom burden in the current clinical setting, and the demand for alternative methods for monitoring disease-specific symptoms has seen an acceleration in recent years. Smartphone technologies offer the potential for continuity of care and surveillance. As a part of a quality improvement project, a disease specific app was developed in collaboration with a software company (Health Touch Ltd) and made available for patient use in May 2018. The Fabry mobile app records five categories: pain, gastrointestinal symptoms, sweating, activity levels, medications. Fabry disease patients with gastrointestinal and pain symptoms attending the Lysosomal Storage Disorders Unit of the Royal Free London NHS Foundation Trust were reviewed to assess eligibility and invited to download the app for recording their symptoms (activity, sweating, pain and gastrointestinal) and medications. Patient-generated data were transmitted to a secure website for clinicians to review.

**Results:**

One-hundred and thirty-nine symptomatic Fabry disease patients who had a smartphone (iPhone or android) were invited to download the app. Sixty-seven patients (26 males and 41 females; median age, 49 years [range, 20–81]) downloaded and tracked the Fabry App at least once. The median frequency of use per patient was 6 (range, 1–629). Pain in the hands and abdominal pain were significantly higher (*p* = 0.009 and *p* = 0.007, respectively) in patients with classic phenotype compared with patients with non-classic phenotypes.

**Conclusions:**

We demonstrated the feasibility and acceptability of a smartphone app to facilitate the remote assessment and monitoring of Fabry disease symptom burden on a daily/weekly basis, as an alternative to the current standard of care that requires patients to recall their symptoms during 6 to 12 monthly annual clinic visits. Patients who were more likely to use the app had greater disease burden. This innovation has the potential to assess disease progression, early therapeutic intervention, thereby decreasing the burden of morbidity and mortality among Fabry patients, and to record long-term effects of Fabry-specific therapies.

**Supplementary Information:**

The online version contains supplementary material available at 10.1186/s13023-023-02999-6.

## Background

Fabry disease (OMIM 301500) is a progressive x-linked inherited disorder caused by pathogenic mutations in the *GLA* gene leading to deficient or absent α-Galactosidase A (α-Gal A) activity [1, 2]. Depending on the mutation, the enzyme activity may be reduced or abolished. Deficiency in the α-Gal A activity leads to a progressive intracellular accumulation of glycosphingolipids, mainly globotriaosylceramide, in the lysosomes of various cell types throughout the body [2–4]. Glycosphingolipid storage initiates a cascade of events, beginning with the dysfunction of basic metabolic processes at cellular level and progressing to cell death and inflammatory events that ultimately lead to irreversible organ damage affecting kidneys, heart and nervous system [3, 5].

Fabry disease is characterized by a large genotypic and phenotypic spectrum: the two most common clinical phenotypes are the classic and non-classic phenotype (often referred to as later-onset or atypical form) [2]. In the severe classic form, signs and symptoms of Fabry disease typically emerge in childhood or adolescence and include neuropathic pain, primarily acroparesthesia (i.e. burning sensation in the hands and feet) and sweating abnormalities (i.e. hypohidrosis or anhidrosis), and gastrointestinal problems (i.e. abdominal pain, nausea, vomiting, bloating, and alternating episodes of diarrhoea and constipation related to gastrointestinal dysmotility caused by autonomic dysfunction), which can substantially influence health-related quality of life [6–8]. Proteinuria, electrocardiographic changes and retinal vessel tortuosity have also been recently described in childhood Fabry disease [9–11]. In adulthood, Fabry manifestations cause early and significant cardiac, renal and cerebrovascular manifestations [12]. In contrast, the less severe later-onset variants are characterized predominantly by asymptomatic childhood and a progressive development of cardiac, renal and/or cerebrovascular manifestations in adulthood [12].

Current healthcare needs are based on intermittent, face-to-face monitoring of Fabry symptoms and signs at clinic appointments every 6–12 months; however, Fabry symptoms are often variable and difficult to monitor, in particular the gastrointestinal and pain symptoms as well as their impact on day-to-day living. There are various quality of life questionnaires, including a disease specific health- and pain-related questionnaire for paediatric Fabry disease [13], which is completed by patients at clinic visits only—usually once every 6–12 months—and is considered a reliable tool for monitoring pain and optimising its management. However, when patients are reviewed once or twice a year it can be difficult to get a detailed picture of their symptoms since the last assessment.

Mobile phones and tablets are widely used, with an estimated median of 76% of the population across advanced economies, including children, regularly using these devices [14]. Increasing number of mobile phones is now classed as smartphones, with capabilities of using applications or apps. Recent advances in portable technology, such as the development of mobile health apps, make smartphone ideal tools to increase peoples’ engagement in their own health and lifestyle management, and—in partnership with heath care professionals—the opportunity to personalise and tailor patient’s care and for continuous monitoring thus offering a potential to optimise treatments. Such tools are currently being used in various health care settings, such as general practitioner surgeries and hospitals for monitoring diabetes and liver transplant patients respectively [15, 16].

**Fig. 1:**
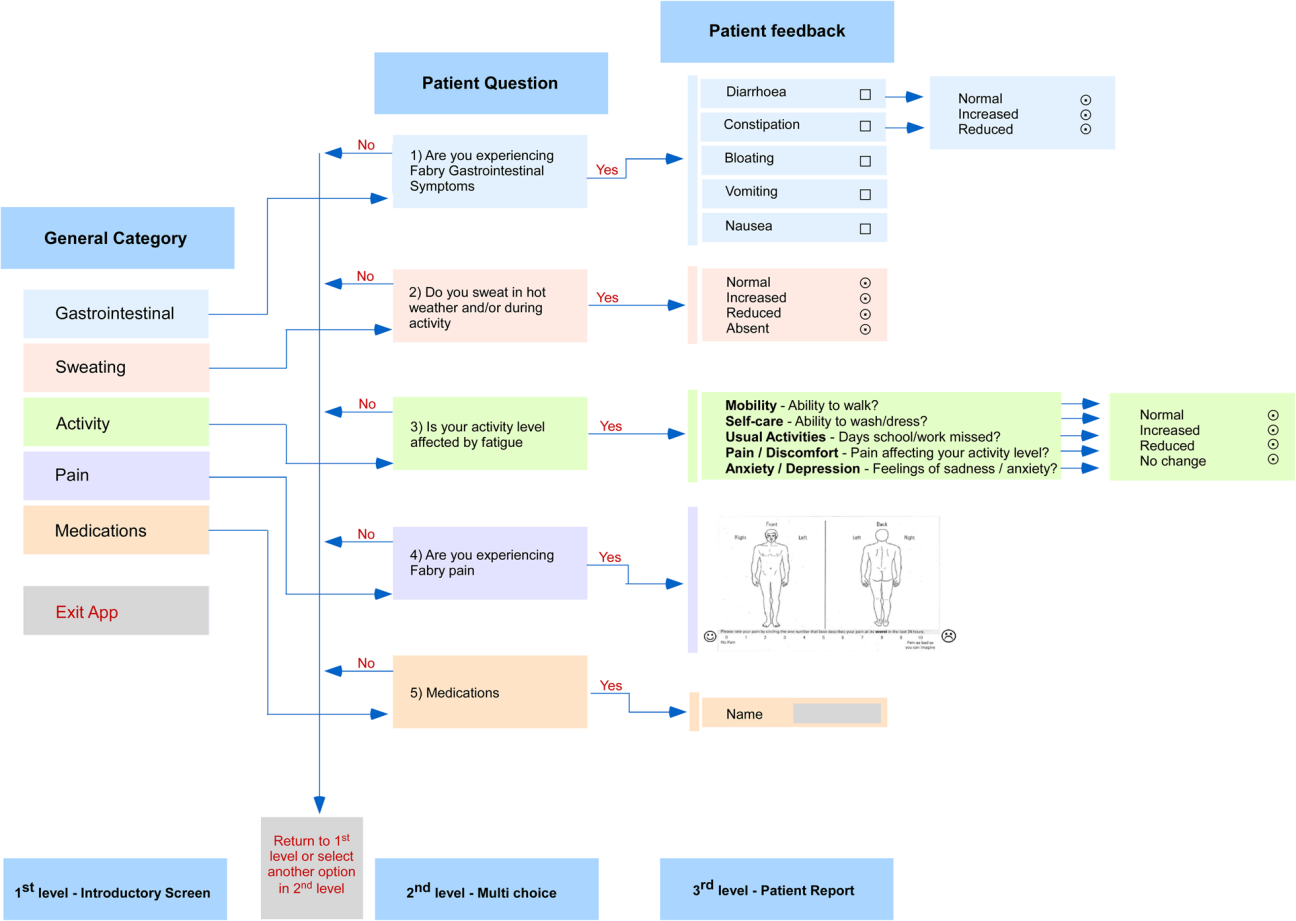
Fabry App development process. The figure summarises the development process and design of the Fabry App

**Fig. 2:**
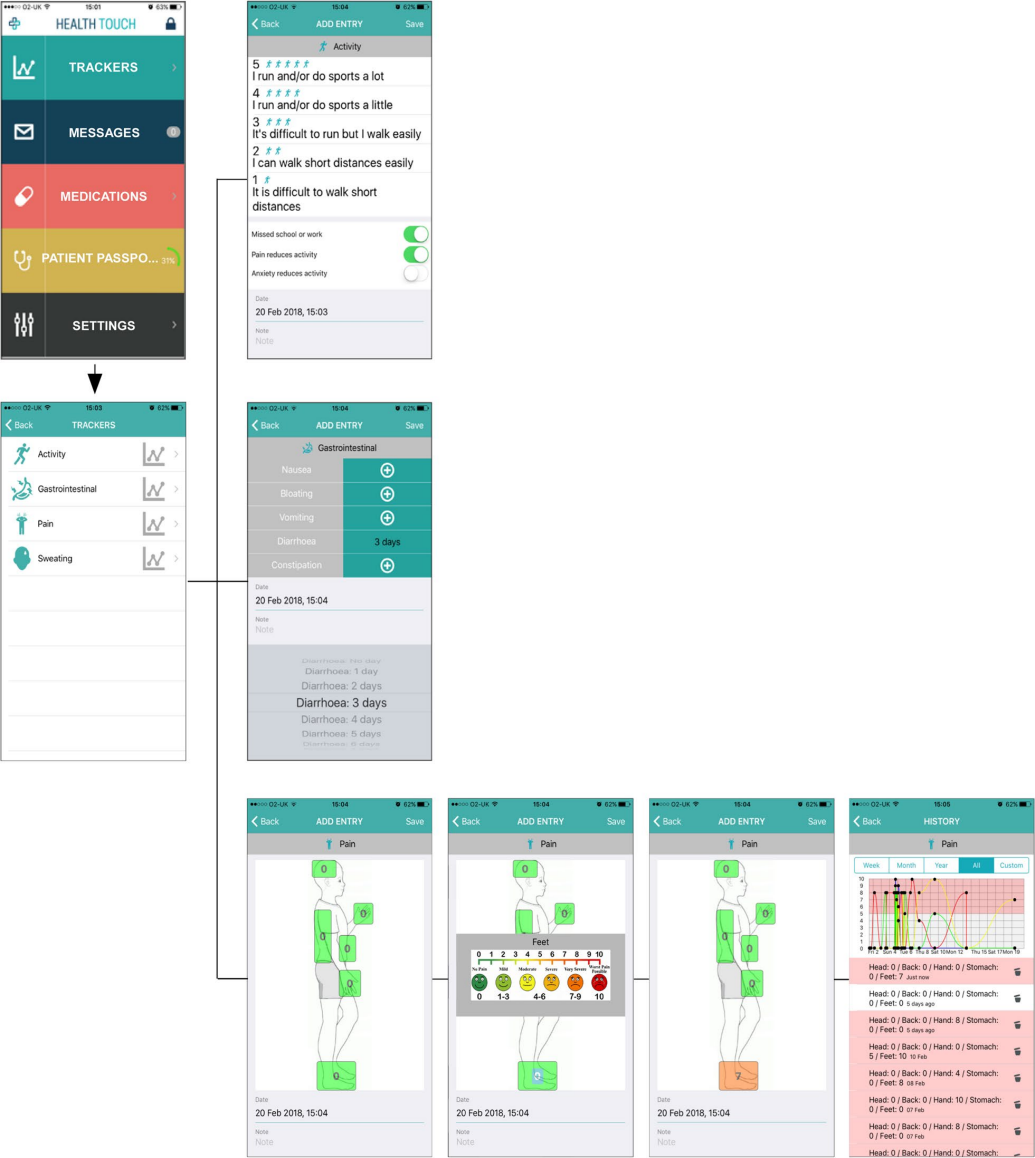
Overview of the Fabry App. The figure shows the app launch screen with options to enter data into ‘Trackers’, read/write ‘Messages’, read ‘Medications’, enter ‘Patient Passport’ information and adjust ‘Settings’. Via ‘Trackers’ the patient can choose from a category of symptoms (activity, gastrointestinal, pain, sweating). The patient is asked to enter tracker information on a weekly basis. He/she is also asked to complete and submit a Quality of Life form every month via the app

In partnership with Health Touch Ltd, a software company that uses web and mobile technologies to bring together healthcare professionals and patients, we developed a bespoke Fabry App to provide patients with the opportunity of monitoring the day-to-day variations in their symptoms and of being able to view changes over a period of time, and clinicians with the possibility of a regular, remote monitoring of symptoms to gain a better understanding of longer-term symptom management and treatment effects. As part of this, we conceived and developed a unique pain tracker which includes a visual analogue scale (Figs. 1, 2). In this quality improvement project, our main aims were (1) to evaluate whether Fabry disease patients would download and use the app; and (2) to evaluate their engagement in using the app thereby enabling health care providers to capture changes in their symptoms.

## Methods

### Design

We conducted a feasibility project of a mobile app as part of routine service evaluation, designed for the management of pain and gastrointestinal symptoms in patients with Fabry disease. Patients were consented and instructed in the use of the app during their routine clinic appointment. Symptoms and medications were reviewed at outpatient clinical appointment. Patients and healthcare providers were also interviewed for feedback to obtain qualitative data.

### The technology platform—health touch

Health Touch is a cloud-based application that can be downloaded to any Apple or Android phone or device. “Cloud” computing means that data are stored in an online secure location. This data is accessible from any computing device to those with the correct authority to access this information safely, via the use of password protection. The Health Touch platform is a Registered MHRA Class 1 non-measuring device. Data is protected by user ID and password in the mobile app and is also protected in transit using transport layer security (TLS) encryption and protected in storage by encryption and strong password protection, housed in facilities using physical security measures. Health Touch Ltd also works to a clear, documented privacy policy and conforms to the NHS Data Protection Toolkit standards.

Health Touch has two parts: a simple and accessible app for patients to record key stats and medications and a website for clinicians to review patients home monitoring data as charts, forms and history.

Entering trackers is quick and simple for patients. They receive reminders to track and can see messages from clinicians.

Clinicians can set thresholds for patient symptoms, set tracking reminders, send messages and see patients’ medications via text and photos. Clinicians can also monitor tracking adherence in addition to symptoms via the website.

Patients consent to share data with the service. The consent message is clear and simple; patients must actively agree for their data to be shared.

### The Fabry App

The Royal Free lysosomal storage disorders unit (LSDU) lead, Uma Ramaswami, conceived the idea of the app and, together with Mark Mckie, collaborated with Health Touch team to design additional screens and functions to create the Fabry App, based on the Health Touch platform.

**Fig. 3:**
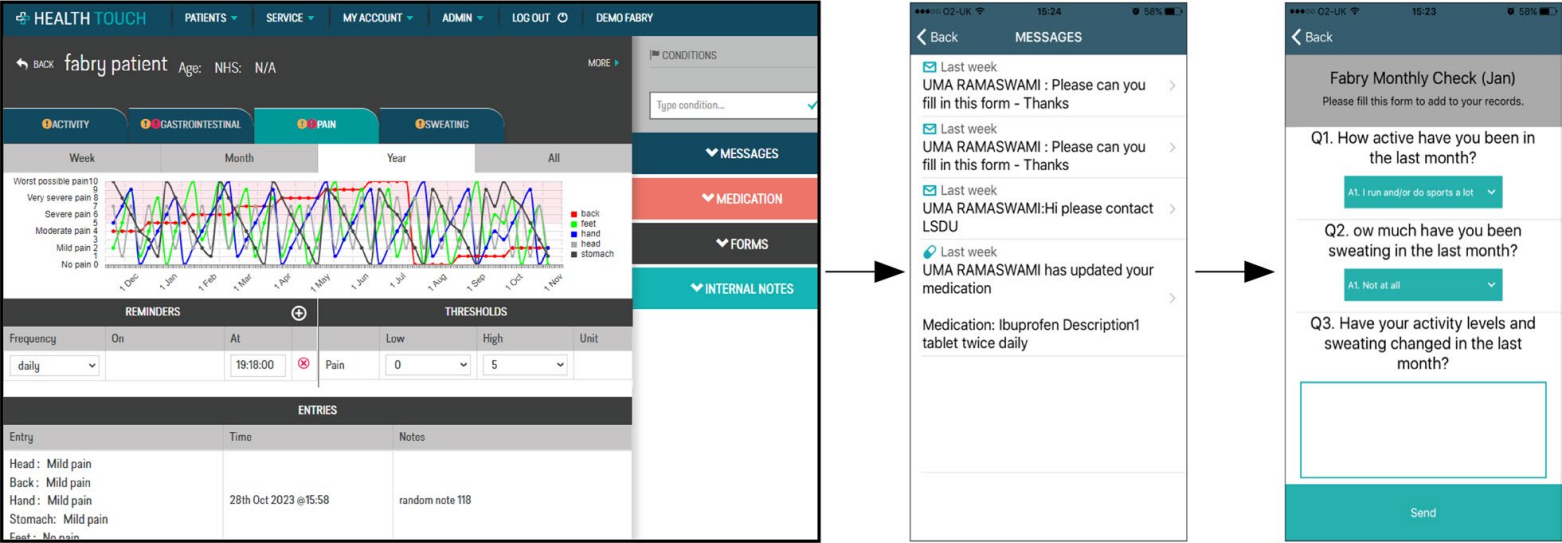
Clinician portal. Patient information is transmitted electronically to the secure website for clinicians to review patients home monitoring data. The clinician can see the tracker information, monthly Quality of Life form and messages via website. Thresholds can be set, tracking reminders sent and medications entered. Data can be seen in graphical format as well as exported in a table format for analysis

The Fabry App is a simple, accessible portable technology that allows patients to record key trackers (i.e., activity, gastrointestinal symptoms, pain, sweating) and medications (see Figs. 1, 2, 3, and Additional file 1: Appendix S1 for details). This disease specific app has been developed using published and routinely used questionnaires, such as the Fabry specific paediatric health and questionnaire (FPHPQ) [13] and Royal Free Fabry assessment questionnaire (Additional file 1: Appendix S2).

The information provided by patients is transmitted electronically to a secure, password protected website for medical professionals to review the patients home monitoring data. The data is secured by encrypting, (i.e., converting information or data) into a code, especially to prevent unauthorised access.

A data protection impact assessment (DPIA) was carried out by the Information Governance Team of the Royal Free London NHS Foundation Trust to identify potential privacy risks and explore the potential impact of this service improvement on the data subject’s right, and to ensure its compliance with the requirements of the Data Protection Act and general data protection regulation (GDPR). At all levels, the security and confidentiality of the information and the patient has been approved and comply with the Data Protection Legislation.

### Patients

A screening questionnaire (Additional file 1: Appendix S2) with questions to ascertain eligibility was administered to 400 patients with a confirmed diagnosis of Fabry disease attending the LSDU at the Royal Free London NHS Foundation Trust (RFL) in 2018. Screening questionnaires were then reviewed to find suitable candidates. Patients who had smartphones (either iPhone or Android) and who had gastrointestinal and Fabry-related pain symptoms were invited to download the Fabry App after providing informed consent. The patients included those diagnosed with classic and non-classic disease variants. As this was a pilot quality improvement project, we did not have the resources to develop the app in different languages other than English; however, the app was designed with an intuitive and easy-to-use interface, and with powerful visual cues to help users navigate it effectively, including non-native speakers (Fig. 2).

Once recruited, patients received phone calls from the Lysosomal Storage Disorders Unit team to assess progress and remind patients to use the app.

### Data

Data on symptoms and medications were entered by patients using the app and transmitted electronically to a secure website for clinicians to review. The downloadable patient monitoring data were reviewed by the clinical team on a regular basis between clinic appointments to evaluate the impact and/or worsening of pain and gastrointestinal symptoms, and its impact on the patients’ quality of life. The clinical team could contact the patient if there were concerns in between clinic appointments either via message through the app or by telephone call, and the patient reported outcomes were also discussed during their follow-up appointment (face-to-face or telephone clinic) every 6–12 months. However, since the app was conceived to monitor day-to-day variability and not designed to answer to emergency queries and/or to assist patients with health conditions requiring urgent medical attention, patients could contact the clinical team only via telephone call and not via message through the app: this was clarified to patients to ensure a proper use of the app and that no urgent or emergency calls from patients were missed.

### Statistical analysis

A formal sample size calculation is not required for pilot and feasibility studies [17]. Descriptive data are described as number and percentage for categorical variables, and median (range) for continuous variables. Two-sample t-test was conducted to investigate differences between patients with classic phenotype and those with non-classic phenotype. All the statistical tests were performed using the NCSS software (Kaysville, UT, USA); the null hypothesis was rejected when the *p* value was ≤ 0.05.

## Results

### Patient characteristics

After screening 400 consecutive adult Fabry patients attending the LSD clinic, 139 patients who had pain and/or gastrointestinal symptoms and owned a smartphone were invited to participate to the study. Of these, 72 (52%) did not download/track the Fabry App, while the remaining 67 (48%) used it at least once. Patients who did not use the Fabry App had a median age of 46 (range 17–77) years, 23 had a clinical diagnosis of classic phenotype (7M:16F) and 41 had a diagnosis of non-classic phenotype (22M:19F). Patients who used the Fabry App had a median age of 49 (range 20–81) years, 17 (7M:10F) had classic phenotype and 50 (19M:31F) had non-classic. The majority of patients who used the Fabry App were on Fabry-specific treatments (28 on enzyme replacement therapy; 21 on oral chaperone; and 1 on other treatment), while 17 were not on any Fabry-specific treatment. Baseline characteristics of participants are summarised in Table 1.

**Table 1:** Patient characteristics

Characteristics	
Age (years), median (range)	49 (20–81)
*Gender, n (%)*	
M	26 (39)
F	41 (61)
*Clinical phenotype, n (%)*	
Classic	17 (25)
Non-classic	50 (75)
*Treatment status, n (%)*	
Enzyme replacement therapy	28 (42)
Oral chaperone	21 (31)
Other treatment	1 (2)
No on Fabry-specific treatments	17 (25)

### Mobile device-related data

Data entered between May 2018 and December 2022 were reviewed. During this period, the median frequency of use of the Fabry App was 6 entries per patient (range, 1–629); a slightly higher frequency of use was observed in patients with classic phenotype (median entries, 6 [range, 1–629]) compared with those with non-classic phenotype (median entries, 5 [range, 1–120]). Forty-four patients (9 [4M:5F] with classic phenotype and 35 [13M:22F]; 11 [25%] were not on Fabry-specific treatments) had at least one entry per month, 16 (6 [1M:5F] with classic phenotype and 10 [6M:4F] with non-classic variant; 4 [25%] were not on Fabry-specific treatment) had two or more (up to 34) entries per month. The remaining 7 patients (2 male patients with classic phenotype and 5 [1M:4F] with non-classic variant; 2 (29%) were not on Fabry-specific treatment) tracked the app only once and had mild Fabry symptoms as shown by a median activity score of 4 (range, 1–4), a median sweating of 2 (range, 0–2), the absence of pain in their hands, feet and stomach (only two patients reported back and head pain) and the gastrointestinal symptoms that were either absent (nausea, vomiting, constipation) or mild (median of 1 for bloating and diarrhoea). In the overall population, the median monthly use of the Fabry App was 2 entries (range, 1–18) and the median time between entries was 16 days (range, 0–438).

Interestingly, patients with classic phenotype showed higher pain scores in their hands compared with those with non-classic phenotype (median score per patient, 4 [range, 0–8] and 0 [range, 0–7]; *p* = 0.009) and abdominal pain (median score per patient, 2 [range, 0–8] and 0 [range, 0–8]; *p* = 0.007). Patients with classic phenotype also reported slightly higher intensity of pain in other sites compared with those with non-classic phenotype, such as feet (median score per patient, 1 [range, 0–8] and 0 [range, 0–9]; *p* = 0.285), back (median score per patient, 3 [range, 0–8] and 0 [range, 0–10]; *p* = 0.119) and head (median score per patient, 3 [range, 0–8] and 1 [range, 0–10]; *p* = 0.369), although there was not a statistically significant difference between the two groups, due to small numbers who reported the symptoms.

Patients with classic phenotype showed slightly higher occurrence of gastrointestinal symptoms, compared with those with non-classic phenotype, such as bloating (median score per patient, 1 [range, 0–4] and 0 [range, 0–7]; *p* = 0.283), constipation (median score per patient, 1 [range, 0–4] and 0 [range 0–5]; *p* = 0.155) and diarrhoea (median score per patient, 1 [range, 0–5] and 0 [range, 0–7]; *p* = 0.252). Nausea and vomiting were less frequently reported in both classic (median nausea score, 0 [range, 0–3]; median vomiting score, 0 [range, 0–0]) and non-classic phenotype patients (median nausea score, 0 [range, 0–4]; median vomiting score, 0 [range, 0–1]). However, none of the gastrointestinal symptoms was statistically significantly different between the two groups due to small numbers who reported the symptoms.

Patients with non-classic phenotype recorded slightly higher levels of activity (median score per patient, 3 [range, 0–5]) compared with patients with classic phenotype (median score per patient, 2 [range 0–5]), although there was not a statistically significant difference between groups (*p* = 0.305). Notably, we observed that both patients with classic phenotype and those with non-classic phenotype reported absent or very little sweating (median score, 0 [range, 0–4] in both groups; *p* = 0.944).

### Patient notes

The Fabry App also offers the possibility to patients to record notes thus providing the clinician with additional information regarding intercurrent illness (e.g., influenza, SARS-CoV-2 infection, etc.) and medications (e.g. newly prescribed or discontinued drugs) as well as with a qualitative description of their complaints thus facilitating the clinical interpretation of their symptoms as we all as try to better understand their burden in terms of impact on patient’s life (e.g., days missed at school/University or work). The Fabry App also revealed its potential for remote patient monitoring during the Covid-19 pandemic when disruptions occurred across the Lysosomal Storage Disorders services in the United Kingdom. In fact, during the first wave (March 2020–June 2020) a treatment interruption of 12 weeks was offered to those patients on enzyme replacement therapy with home care nursing support as the risk of contracting SARS-CoV-2 was considered to outweigh the risk of treatment interruption. While treatment interruptions did not result in any major event nor disease progression, several Fabry patients reported an increase in fatigue and low energy levels as highlighted by the virtual consultations conducted during that period by our team [18]: these symptoms were not only captured by the Fabry App but also accompanied by in-depth descriptions of their characteristics as well as by specific social and/or psychological needs (e.g., anxiety management).

### Patient and LSD provider feedback

Qualitative feedbacks were obtained from clinicians and patients to review the psychological impacts of such digital health solution. Patient’s comments were overall very positive as the app was perceived as easy to use and very accurate to track symptoms. Patients reported a better sense of disease status. None of the participants felt that the app violated their personal privacy.

Patient A, a female patient with Fabry disease, said she ‘was up for the idea’ as soon as she heard about the app. Ellen said: “My Fabry’s can be so up and down so being able to record my symptoms and levels of pain like this is really useful. It’s been great because my consultant can follow my progress and make recommendations about my treatment or the support I need, which can make a real difference to my health and how I feel.”

Patient B, a male patient with Fabry disease, said: “Up until now you’ve had no way of feeding back what’s been going on between the six-month appointments. I jumped at the opportunity as I am a data science nerd!”.

“I do a weekly update on the app. Over 40 years I’ve got to know my triggers pretty well and I know what to do to avoid major flare ups, but it’s a difficult disease and it is up and down. Now I can meet with my consultant, and we can look over the data together and I can explain what was going on when my symptoms got worse.”

“I think that the exciting thing is there’s now a cohort of Fabry patients all over the country putting in their data and this will be a great resource to help the team to find ways to help patients further and I hope it might also show if other people get the same kinds of reactions or symptoms as I do. I’d be really interested to know for instance if other people experience pins and needles in their gums like I do.”

“The doctors and nurses have all been solid gold. It took till my 40s to find out what the matter was with me as I was adopted so there was no family history to help signpost but ever since I got my diagnosis, I couldn’t have asked for better care.”

According to the clinicians who reviewed the symptoms recorded in the Fabry App during the clinical appointments (outpatient and telephone), the app facilitated the monitoring of response to treatment as it provides patients with the opportunity to report symptoms differently without trying to remember their severity over a prolonged period of time and it takes only a few minutes to input. By providing instant access to drug information, the Fabry App is also useful for keeping track of other medications beyond Fabry-specific treatments, including drugs prescribed by other physicians if a patient had added them, and intervene in case of side effects. As an example, the Fabry App facilitated the diagnosis of opioid-induced constipation in a patient with acroparaesthesia who had increased use of opiates for pain relief: as a result, the patient was contacted, and pain medications reviewed. Similarly, other patients showing increased scores for pain, were contacted by the clinical team to investigate the underlying cause (e.g., Fabry-related pain, functional pain associated with psychological issues, etc.) and review their pain medications accordingly.

## Discussion

Advances in smartphone technology has had a significant impact in medicine: it has allowed the integration of digital technologies including the use of mobile devices into routine clinical practice. A number of apps have been developed in recent years to monitor chronic conditions such as cancer [19, 20], cirrhosis [21], chronic obstructive pulmonary disease [22], diabetes [23, 24], rheumatoid arthritis [25], depression [26] among others, as well as—most recently—to support the monitoring and management of acute medical conditions such as SARS-CoV-2 infection [27, 28]. Most individuals living in the United Kingdom own and use smartphones and/or tablets, which increases the feasibility of using mobile health apps to monitor and manage chronic diseases. However, while in recent years there has been an exponential increase in apps designed to track and monitor patients with chronic conditions, apps for Fabry disease are missing and current recording of patient’s disease symptoms occurs only at clinic appointments every 6–12 months. We therefore designed a mobile app to monitor day-to-day variations in symptoms and their effects on quality of life of individuals with Fabry disease. The app was made available for patient use in May 2018: it allows patients to monitor their health in a convenient setting of their choice. It also enables health care professionals to have improved and regular monitoring of patient’s symptoms, instead of the snapshot review at a clinic appointment once every 6–12 monthly, relying solely on patient recall of their symptoms during the prior 6–12 months. This quality improvement project’s primary goal was to evaluate whether Fabry disease patients would be willing to use the app and to confirm its value in a clinical setting. This project revealed a greater engagement of those patients severely affected by Fabry disease, who therefore may be identified as the “ideal patient” for being supported by the app: a possible explanation may be that patients with symptoms affecting their quality of life are more inclined to document their symptoms via the app compared with patients who are less symptomatic or asymptomatic who tracked the app only once as shown by our results.

To the best of our knowledge, this is the first mobile app designed for monitoring the symptomatology and quality of life in Fabry disease patients.

### Strengths and limitations

This was a service improvement evaluation that aimed to explore the feasibility of a new health monitoring system designed for Fabry disease patients to improve their healthcare delivery. These results indicate that a well-designed app can empower Fabry disease patients and help the clinician to closely monitor changes of their symptoms. It has also helped us in identifying the strengths and limitations of the Fabry App. Strengths of the app are represented by the stakeholders’ involvement (healthcare staff and patients) during the app development aimed to identify potential barriers to and facilitators of using the app. One of the limitations identified is the need for resources for dedicated staff reviewing the data on a regular basis thus allowing more robust monitoring and for the maintenance of the app to avoid technology issues. Another limitation is the lack of motivation in some patients that choose not to use the app or only used it for a short period: this was due to being less symptomatic or asymptomatic rather than concerns for their health information security and privacy as confirmed by the milder/absent symptoms recorded by those who tracked the app only once in comparison with more symptomatic patients who recorded their symptoms regularly. In this light, wearable sensor technologies, which enables detection of health-state parameters (such as heart rate, breathing rate, body temperature, blood oxygen saturation, position, activity during the day by counting steps and posture), have the capacity for continuous data recording coupled with a potential larger use due to better compliance. Finally, the Fabry app was designed to capture subjective symptoms of Fabry disease with a particular focus on activity levels, sweating, pain and gastro-intestinal manifestations and their impact on patient’s quality of life and not for monitoring vitals nor objective symptoms (i.e., shortness of breath/dyspnoea, palpitations, chest pain, etc.) that would require a constant monitoring (24/7) of a fully dedicated and trained health staff for ensuring proper assistance in case of acute deterioration requiring urgent medical attention. This aspect was clarified to patients to ensure a correct use of the app and within its scope.

## Conclusion

In conclusion, this service improvement supports the feasibility of a smartphone app to monitor Fabry disease. Fabry disease patients reported that that the mobile app was easy to use, and clinicians found it helpful when reviewing patients in clinic and appreciate an increasing patient’s engagement in their own health and lifestyle management.

Our ultimate objective is to improve the clinical outcomes for Fabry disease patients by providing greater quality of care and, most importantly, by empowering patients to take charge of their own health.

### Supplementary Information

**Additional file 1: Appendix S1** List of symptoms available on the Fabry App for patients to choose from and submit to the Lysosomal Storage Disorders team; and **Appendix S2** Use of Phone App: Screening Questionnaire.

## Data Availability

The raw datasets used and/or analysed during the current project are not openly available due to reasons of sensitivity to preserve individuals’ privacy under the European General Data Protection Regulation. Only excerpts of the datasets are available from the corresponding author (uma.ramaswami@nhs.net) on reasonable request.
