# Supplementary material for: Fabry App: the value of a portable technology in recording day-to-day patient monitored information in patients with Fabry disease
Source: Orphanet J Rare Dis. 2024 Jan 11;19:13. doi: 10.1186/s13023-023-02999-6 (PMC11057153; doi:10.1186/s13023-023-02999-6)
Supplement: Supplementary file 1 — Additional file 1: Appendix S1 List of symptoms available on the Fabry App for patients to choose from and submit to the Lysosomal Storage Disorders team; and Appendix S2 Use of Phone App: Screening Questionnaire. [file 13023_2023_2999_MOESM1_ESM.docx]

**Fabry App: the value of a portable technology in recording day-to-day patient monitored information in patients with Fabry Disease**

**Journal of Medical Systems**

Simona D’Amore^1^, Mark Mckie^1^, Andrew Fahey^2^, David Bleloch^1^, Giuseppina Grillo^1^, Michael Hughes^2^, Uma Ramaswami^1*^

^1^Lysosomal Storage Disorders Unit, Royal Free Hospital NHS Foundation Trust London, United Kingdom

^2^ HealthTouch Ltd, Little Halt, Bull Lane, Chislehurst, Kent, United Kingdom

***CORRESPONDENCE**

Dr Uma Ramaswami FRCPCH, MD

Consultant in Inherited Metabolic Disorders

Lysosomal Disorders Unit

Royal Free London NHS Foundation Trust

Pond Street, London NW3 2QG, United Kingdom

Email: uma.ramaswami@nhs.net; ORCID: 0000-0002-4703-7447

**Appendix 1. List of symptoms available on the Fabry App for patients to choose from and submit to the Lysosomal Storage Disorders team.**

| **Symptoms** |  |
| --- | --- |
| Pain | *Intensity (0-10):*  No 0  Mild 1-2  Moderate pain 3-4  Severe pain 5-6  Very severe pain 7-8  Worst possible pain 9-10  *Location:*  Hand, foot, back, stomach, head |
| Gastrointestinal | *Symptoms:*  Nausea  Bloating  Vomiting  Diarrhoea  Constipation  *Frequency 0-7 days in week* |
| Activity | *Intensity 1-5*  Difficult to walk short distance 1  Walk short distance easily 2  Difficult to run but walk easily 3  Run and/or do sport a little 4  Run and/or do sport a lot 5 |
| Sweating | *Intensity (0-4):*  None 0  A little 1  Some 2  Lots 3  Always 4 |

**Appendix 2. Use of Phone App: Screening Questionnaire**

**To be on NHS headed paper**

Dear Patient,

I am writing to invite you to take part in an exciting project involving the use of a portable device, such as a mobile app, to capture day to day variations in your/ your child’s symptoms. At present there are limited resources for us to assess the day to day affects Fabry disease has on you. Hopefully the use of a mobile app can record the data needed to assess the symptoms and capture the real-life burdens of Fabry disease, which will possibly help the way we personalise treatments and control symptoms in the future.

Please complete the following Screening Questions by initialling the correct boxes and return in the self-addressed envelope:

**YES NO**

Do you suffer from Fabry related Pain?

Do you suffer from Fabry relayed Gastrointestinal Symptoms?

Are you currently receiving Enzyme Replacement Therapy?

Do you have access to a Smart Phone?

Would you be interested in participating in the study explained in this letter and the

information sheet attached?

**Signature**: **Date**:

**Name of Patient**
